# Isoorientin ameliorates H_2_O_2_-induced apoptosis and oxidative stress in chondrocytes by regulating MAPK and PI3K/Akt pathways

**DOI:** 10.18632/aging.204768

**Published:** 2023-06-05

**Authors:** Tiehan Cui, Yun Lan, Yuying Lu, Fei Yu, Suai Lin, Yizhe Fu, Jiaxuan Qiu, Guangliang Niu

**Affiliations:** 1 Department of Oral and Maxillofacial Surgery, The First Affiliated Hospital of Nanchang University, Nanchang 330006, China; 2 Department of Stomatology, Beijing Hospital of Integrated Traditional Chinese and Western Medicine, Beijing 100039, China; 3 Medical Innovation Center, The First Affiliated Hospital of Nanchang University, Nanchang 330006, China

**Keywords:** isoorientin, RNA-seq, bioinformatics, apoptosis, oxidative stress

## Abstract

Osteoarthritis (OA) is a chronic and complicated degenerative disease for which there is currently no effective treatment. Isoorientin (ISO) is a natural plant extract that has antioxidant activity and could be used to treat OA. However, due to a lack of research, it has not been widely used. In this study, we investigated the protective effects and molecular mechanisms of ISO on H_2_O_2_-induced chondrocytes, a widely used cell model for OA. Based on RNA-seq and bioinformatics, we discovered that ISO significantly increased the activity of chondrocytes induced by H_2_O_2_, which was associated with apoptosis and oxidative stress. Furthermore, the combination of ISO and H_2_O_2_ significantly reduced apoptosis and restored mitochondrial membrane potential (MMP), which may be achieved by inhibiting apoptosis and mitogen-activated protein kinase (MAPK) signaling pathways. Moreover, ISO increased superoxide dismutase (SOD), heme oxygenase 1 (HO-1) and quinone oxidoreductase 1 (NQO-1) and reduced malondialdehyde (MDA) levels. Finally, ISO inhibited H_2_O_2_-induced intracellular reactive oxygen species (ROS) in chondrocytes by activating the nuclear factor erythroid 2-related factor 2 (Nrf2) and phosphatidylinositol 3 kinase/protein kinase B (PI3K/Akt) signaling pathways. This study establishes a theoretical framework for ISO’s ability to inhibit OA *in vitro* models.

## INTRODUCTION

Osteoarthritis (OA) is a chronic, complex disease that is the leading cause of disability in the elderly [1–3]. Cartilage is primarily destroyed and usually accompanied by bone sclerosis and inflammation of the synovium. Although the molecular mechanism of OA is unknown, chondrocyte apoptosis is a key feature of the disease [4]. Apoptosis is strongly linked to mitochondrial dysfunction, which results in excess of reactive oxygen species (ROS), which eventually leads to oxidative stress [5, 6]. Increased levels of oxidative stress have also been discovered in OA patients’ synovial fluid and damaged cartilage, as well as in the cartilage of mice with surgically produced OA [7, 8]. Therefore, inhibiting apoptosis and oxidative stress could be useful in slowing OA progress.

Currently, drug therapy is primarily used to treat the symptoms of OA. For example, nonsteroidal-anti-inflammatory drugs (NSAIDs) can reduce pain, but have no significant effect on oxidative stress, mitochondrial dysfunction, and chondrocytes apoptosis. These medications have serious adverse effects, including gastrointestinal issues and toxicity buildup, which limit their long-term use [9]. Future research trends will emphasize alternative treatments with minimal side effects, and slowing disease progression is critical [10, 11]. As OA is characterized by continuous oxidative damage [6], natural antioxidant plant extracts have beneficial effects with few side effects, attracting the attention of many researchers [12, 13].

Isoorientin (ISO) is a natural flavonoid of luteolin glycosides, found in variety of foods such as *Polygonum orientale* and corn [14, 15]. There are many orthopaedic diseases and chronic diseases that can be treated with ISO-rich plants or drugs in Chinese folk medicine. ISO was shown to have few toxic side effects and to perform a variety of functions, including lowering the risk of developing diseases caused by oxidation and inflammation [16, 17]. Previous research also demonstrated that ISO significantly reduced oxidative stress in the liver and kidney [18, 19]. Moreover, ISO increased antioxidant enzyme activity resisting doxorubicin-induced cardiac injury [20]. A previous study found that ISO improved mitochondrial function by modulating the AMPK/Akt/Nrf2 signaling pathway [21]. Furthermore, ISO protected against oxidative damage, apoptosis, and autophagy [22, 23]. As a result of ISO’s antioxidant properties, these compounds can be used in the development of osteoarthritis medication.

Despite the fact that ISO has a wide range of biological activities, it has received little attention. It is likely due to a lack of ISO research and unclear ISO mechanisms. There have been few studies focusing into the mechanism of ISO protection on primary chondrocytes from a rat knee up until now. A series of experiments were carried out to investigate the mechanism of ISO action on chondrocytes. Firstly, we discovered that ISO could help prevent some of the cell death caused by H_2_O_2_. Secondly, the cell samples were calculated by RNA-Seq analysis integrated bioinformatic analysis. Thirdly, the findings indicated that ISO can reduce apoptosis and oxidative stress via the MAPK and PI3K/Akt signaling pathways. Finally, western blot, flow cytometry, and other experiments were used to validate the results of the preceding analysis. In summary, ISO is a potential OA treatment with low toxicity and anti-inflammatory properties. This work contributes to the study of ISO anti-inflammatory mechanisms and provides theoretical foundation for clinical applications.

## MATERIALS AND METHODS

### Reagents

Dulbecco’s modified eagle medium-F12 (DMEM/F12) and fetal bovine serum (FBS) were purchased from KeyGEN BioTECH (Jiangsu, China) and Cyagen Biosciences, Inc. (Guangzhou, China). Solarbio (Beijing, China) provided cell counting kit-8 (CCK-8), a superoxide dismutase (SOD) kit, malondialdehyde (MDA), and total protein contents (BCA) kits. ROS kit and a MMP assay kit were obtained from Beyotime (Beyotime, China). Rabbit monoclonal antibodies against PI3K, Akt, mTOR, JNK, P38, and ERK, as well as their corresponding phosphorylation antibodies, were purchased from Cell Signaling Technology (MA, US), and apoptosis primary antibodies, including Bax, Bcl-2, Caspase-3, Cleaved Caspase-3, heme oxygenase 1 (HO-1) and quinone oxidoreductase 1 (NQO-1), nuclear factor erythroid 2-related factor 2 (Nrf2), and Keap1 were prepared by Abmart (Shanghai, China).

### Cell culture

Primary chondrocytes are the best cells to study OA because they maintain cartilage structure and function. The primary rat chondrocytes were obtained from iCell Bioscience, Inc. (Shanghai, China) and grown at 37° C with 5% CO_2_ in DMEM/F12 media containing 10% FBS, 1% penicillin, and 1% streptomycin sulfate. Chondrocytes from the second passage were used in the experiments.

### Cell viability assay

### Effect of chondrocytes viability in a variety of concentrations of ISO

After 12 h, chondrocytes were cultured in 96-well plates at a density of 8,000 per well. Following that, chondrocytes were exposed to ISO (0, 5, 10, 15, 20, 25, 30, 35, and 40 μM) for 12 h. The cell viability of chondrocytes was determined using a microplate reader and CCK-8 to measure absorbance at 450 nm (Thermo Varioskan LUX, US).

### Effect of chondrocytes viability treated with ISO and H2O2

A study found that H_2_O_2_ causes *in vitro* inflammation, which could be useful in simulating OA [24]. Eight thousand cells/well of chondrocytes were seeded into a 96-well plate at 12 h. Afterward, chondrocytes were exposed to ISO (5, 20, and 40 μM) for 12 h. The cell media was then treated with 500 μM H_2_O_2_ and allowed to grow for 4 hours. After 30 min of incubation with the CCK-8 solution in each well, the absorbance at 450 nm was measured using a microplate reader (Thermo Varioskan LUX, US).

### Analysis of chondrocytes RNA-Seq transcriptomic data in bioinformatics

TRIzol protocols for RNA extraction and purification from cell samples were carried out in accordance with the manufacturer’s instructions. The total RNA of chondrocytes from the *control group* without treatment, *the OA group* with H_2_O_2_, and *the OA+ISO group* with ISO and H_2_O_2_ was sequenced by Guangdong Magigene Biotechnology Co., Ltd. (Guangdong, China). A fold change threshold of 1.5 and a p-value of 0.05 were used to examine differentially expressed genes (DEGs). The Kyoto Encyclopedia of Genes and Genomes and Gene Ontology (GO) analysis were also used to calculate DEG enrichment, which can be used to obtain information on the fold changes of expressed genes at the molecular level. To determine the metabolic and signaling pathways, the DEGs at various KEGG pathway levels were counted.

### Determination of cell apoptosis

Chondrocytes were subjected to the same procedure as in the previous method. The chondrocytes were stained with an apoptotic dye (5 mL propidium iodide and 195 mL annexin-V binding) and incubated at 37° C in the dark for 15 min. After that, the cell apoptosis rate was determined using a flow cytometer.

### State of mitochondrial membrane potential (MMP) detection

In six-well plates, chondrocytes (1.5 × 10^5^ cells/well) were cultured for 12 h before being treated with ISO at 20 μM and then H_2_O_2_ for 4 h. The medium was then removed and thoroughly cleaned with PBS three times. In a murky atmosphere, chondrocytes were stained with staining solution and incubated for 20 min with JC-1 solution. An inverted fluorescence microscope was used to measure fluorescence intensity at wavelengths of 490/525 nm (green fluorescence) and 525/590 nm (red fluorescence).

### ROS production detection

In six-well plates, chondrocytes were grown at 1.5 × 10^5^ cells per well, followed by 12 h of ISO treatment at 20 μM and then incubation H_2_O_2_ for 4 h. Each well received 100 μL DCFH-DA (10 μM) for 30 min. A fluorescence microscope was used to analyze the fluorescence. Chondrocytes were grown for 12 h in six-well plates at the same concentration as before. Chondrocytes were collected after 12 h of ISO treatment at 20 μM and then 4 h of H_2_O_2_ incubation. DCFH-DA was applied, followed by washing and centrifugation. Flow cytometry was used to assess the level of ROS after following the prescribed procedures (Novocyte, Agilent, US).

### Level of SOD and MDA detection

Eight thousand cells/well of chondrocytes were seeded into a six-well plate at 12 h, followed by 12 h of ISO treatment at 20 μM and then H_2_O_2_ after 4 h of incubation. The cells were collected during the fifth minute of centrifugation. ELISA kits were used to extract supernatants for BCA, SOD, and MDA analysis (as directed by the manufacturer).

### Western blot analysis

Cell proteins were extracted using RIPA buffer and 1 mM PMSF from Beyotime (Jiangsu, China). The lysates were transferred to a PVDF membrane after being electrophoresed by 7.5%–15% SDS-PAGE. The membrane was washed with water after incubating at room temperature for 12 min with 5% skimmed milk powder prepared in TBST solution. A diluted antibody was added and incubated overnight at 4° C. Afterward, the membrane was washed three times with TBST on a horizontal shaker for 5 min each. Following secondary antibody incubation, the membrane was thoroughly washed and imaged on a molecular image. ImageJ software was used to perform statistical analysis on the gray-scale values of the protein bands.

### Statistical analysis

The results from every experiment were run in triplicate and reported as the mean ± standard deviation (SD) (n = 3). The data were computed as the mean ± SD using SPSS 2.0 Software. To compare statistical significance, Tukey’s range test was used. Significant values were defined as those with a p-value of 0.05.

## RESULTS

### ISO recovers chondrocytes viability induced by H_2_O_2_

**Figure 1:**
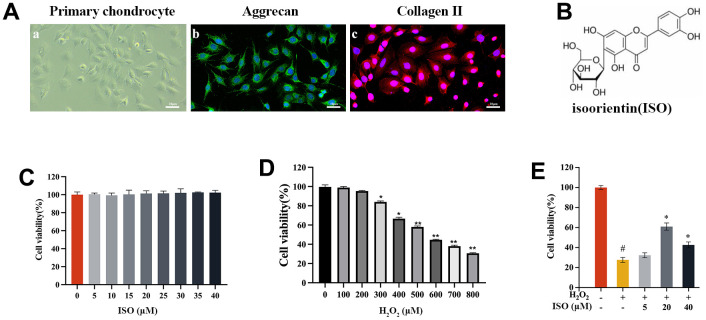
**The effect of ISO or H_2_O_2_ on chondrocytes.** (**A**) Identification of primary chondrocytes in SD rats. (**A**-**a**) shows the morphology of chondrocytes under light microscopy. (**A**-**b**) cellular immunofluorescence detection of ACAN expression. (**A**-**c**) cellular immunofluorescence detection of Col2α1 expression. (**B**) The chemical structure of isoorientin (ISO). (**C**) Toxicity test of ISO on cell viabilities of chondrocytes. (**D**) Effect of different concentrations of H_2_O_2_ on the viability of chondrocytes. (**E**) Effects of ISO on H_2_O_2_-induced injury chondrocytes viability. Results shown are expressed as means ± SD (n = 6). #p < 0.05 compared with control group, *p < 0.05 compared with OA group.

The CCK-8 assay revealed that ISO had no negative effects on chondrocytes within a specified dose range (5–40 μM) (Figure 1C). It was necessary to evaluate the cytotoxicity of H_2_O_2_ in order to determine the appropriate concentration of H_2_O_2_ (500 μM) (Figure 1D). The CCK-8 test was used to estimate cell viability in relation to the protective effect of ISO on H_2_O_2_-induced chondrocytes. Analysis of cell viability revealed that ISO greatly increased cell viability (Figure 1E). After H_2_O_2_ treatment, cell viability decreased to 31% compared to the control group. However, cells pretreated with ISO, particularly at a dose of 20 μM, showed excellent cell survival against H_2_O_2_-induced oxidative damage. Thus, this ISO (20 μM) was used in following studies.

### RNA-seq and bioinformatics analysis predicts ISO potential functions-apoptosis and oxidative stress.

**Figure 2:**
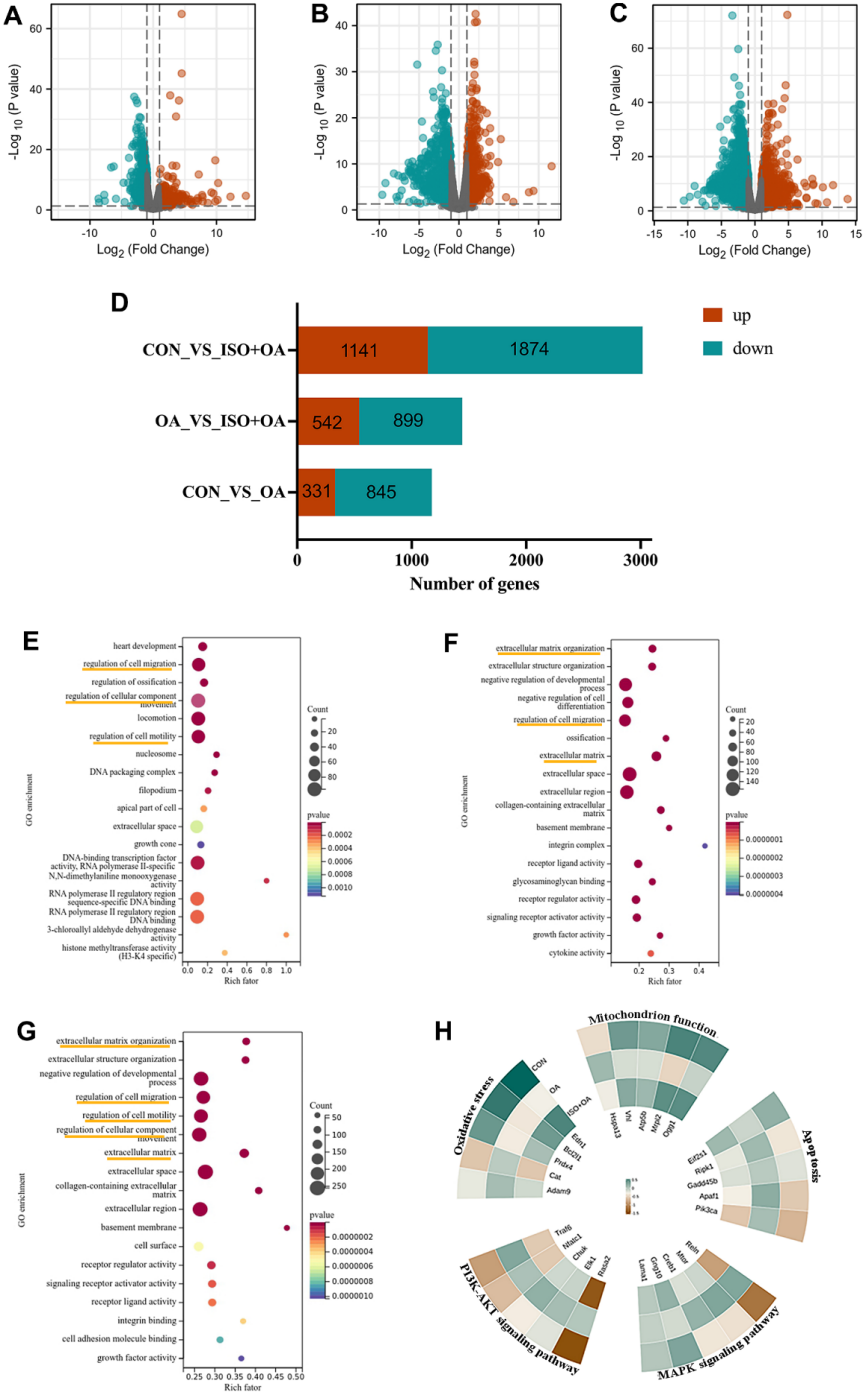
**The result of RNA-seq analysis.** (**A**–**C**) The volcano plot shows the distribution of genes and the results of significant differences in genes. (**A**) Control group vs OA group. (**B**) OA group vs ISO+OA. (**C**) Control group vs ISO+OA. (**D**) The up-regulation and down-regulation of gene in this study, Fold change > 1.5, p < 0.05. (**D**–**F**) The GO enrichment analysis. (**E**) Control group vs OA group. (**F**) OA group vs ISO+OA. (**G**) Control group vs ISO+OA. (**H**) Heat map of the representative function.

**Figure 3:**
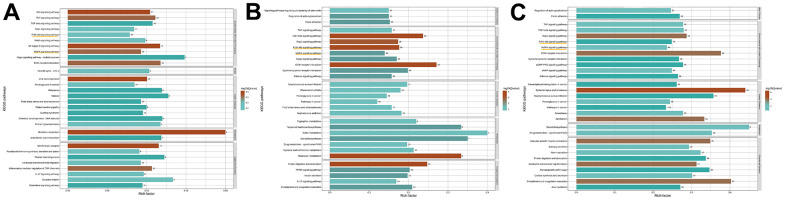
**The result of KEGG analysis.** (**A**) Control group vs OA group. (**B**) OA group vs ISO+OA. (**C**) Control group vs ISO+OA.

After RNA-Seq and bioinformatic analysis, the 542 upregulated genes and the 899 downregulated genes were conducted to identify the mechanism of ISO therapy in H_2_O_2_-induced chondrocytes. A single RNA-seq library represented the transcriptome of each sample. Red denotes upregulated genes, while green denotes downregulated genes, as presented in Figure 2A–2C. The gene change values are shown in Figure 2D. In the model group, a total of 3,015 DEGs were verified, with 1,141 upregulated and 1,874 downregulated compared to the normal group. After ISO pretreatment, 542 genes were upregulated and 899 genes were downregulated in comparison to the OA group. GO analysis of DEGs revealed that “regulation of cell migration”, “regulation of cell component movement”, “regulation of cell motility” and “extracellular matrix organization” were the most significantly altered GO terms (Figure 2E–2G). Among these changes, we summarized the functions of the main changes as “oxidative stress” and “mitochondrion function.” Furthermore, KEGG pathway enrichment analysis (Figure 3A–3C) revealed pathways associated with “apoptosis,” “PI3K/Akt signaling pathway,” and “MAPK signaling pathway.” The genetic changes associated with the aforementioned terms are depicted in Figure 2H.

### ISO protects chondrocytes from apoptosis

### ISO inhibits H2O2-induced chondrocytes apoptosis

**Figure 4:**
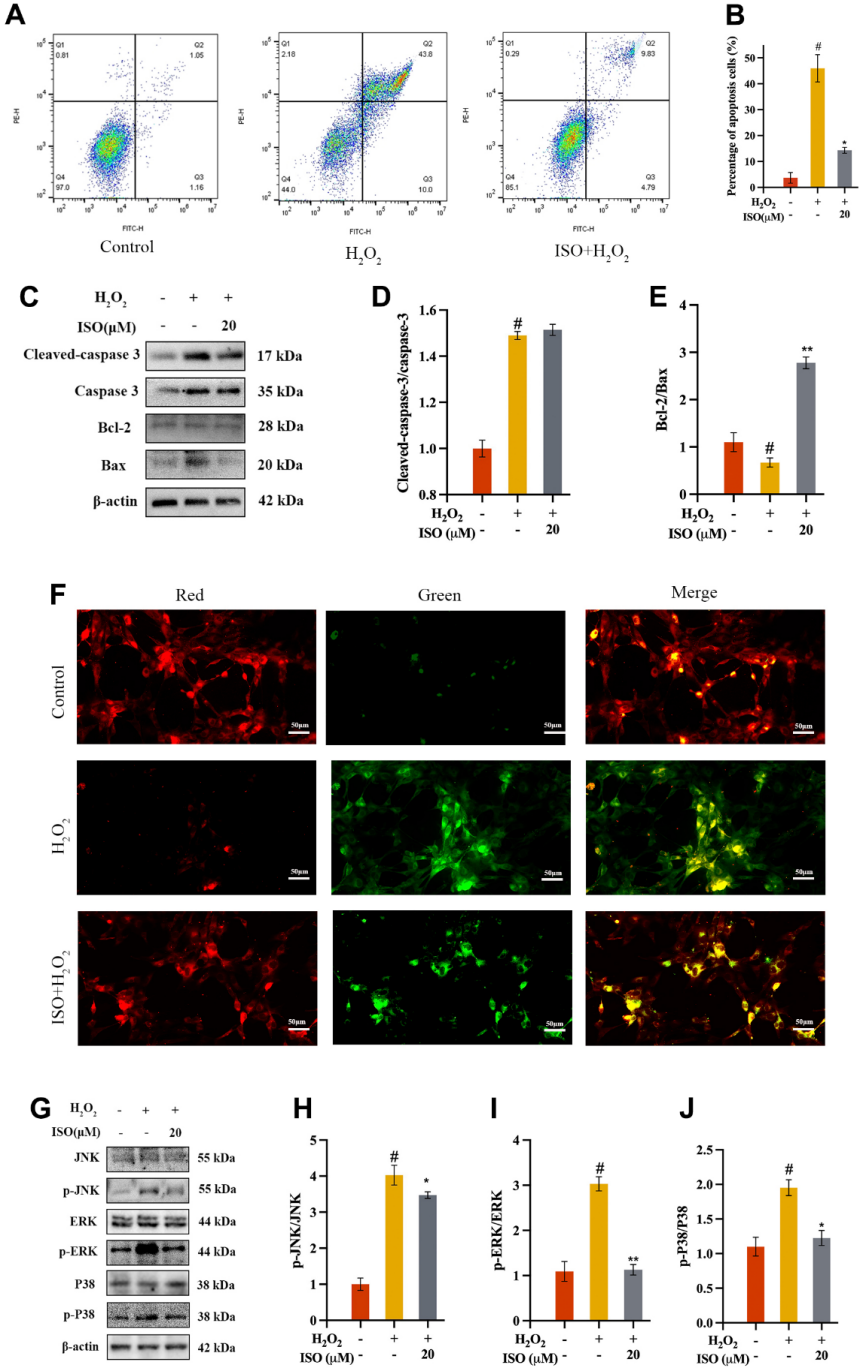
**Effect of ISO on apoptosis.** (**A**, **B**) Effects of ISO pretreatment on the apoptosis by flow cytometry analysis. (**C**–**E**) Effects of ISO pretreatment on the apoptosis pathway by western blotting analysis. (**F**) Effect of ISO pretreatment on the MMP in H_2_O_2_-induced chondrocytes. Red represents high MMP, green represents low MMP. (**G**–**J**) Effects of ISO pretreatment on the MAPK pathway by western blotting analysis. Results shown expressed as means ± SD (n = 3). #p < 0.05 compared with control group, *p < 0.05, **p < 0.01 compared with OA group. The H_2_O_2_ concentration is 500 μM.

Previous research found that ISO inhibited chondrocyte death (Figure 1E). Apoptosis is one of the most common cell deaths induced by H_2_O_2_. According to bioinformatics results, apoptosis was the related death mode that ISO inhibited chondrocyte death (Figure 2H). Additionally, flow cytometry was also used to validate this interaction. As shown in Figure 4A, flow cytometry was performed to determine how ISO affected H_2_O_2_-induced chondrocytes apoptosis. H_2_O_2_-treated chondrocytes produced more apoptotic cells than the control group. It was interesting to note that exposing chondrocytes to ISO reduced the number of apoptotic chondrocytes (Figure 4B), supporting the impact of ISO on chondrocyte apoptosis.

### ISO restores mitochondrion function

Assessment of MMP was used as an indicator of early apoptosis. MMP, caused by an excess of ROS in the mitochondria, destroys DNA and the mitochondrial membrane, resulting in mitochondrial dysfunction [25]. Therefore, MMP was used to assess the protective capacity of ISO against apoptosis by H_2_O_2_ in chondrocytes (Figure 4F). Normal chondrocytes with high MMP fluoresced red after staining with JC-1. However, there was a decrease in membrane potential in the OA group, as evidenced by green fluorescence. The fluorescence returned to almost normal levels in the ISO-pretreated group.

### ISO inhibits apoptosis of H2O2-induced chondrocytes by apoptosis and MAPK signaling pathways

A further attempt to understand its mechanism of ISO inhibiting apoptosis of H_2_O_2_-induced chondrocytes, we validated the key proteins in apoptosis signaling pathways. Key proteins in the mitochondrion were quantified using western blot assays. As a result, mitochondrial proteins (Bax, Bcl-2, caspase-3, and cleaved caspase-3) were identified [26]. The H_2_O_2_-induced more Bax and more cleaved caspase-3 while less Bcl-2 in chondrocytes. The results showed that chondrocytes exposed to ISO had a higher Bcl-2/Bax ratio and lower levels of cleaved caspase-3/caspase-3 (Figure 4C–4E). These findings indicated that ISO regulated the production of the aforementioned proteins in order to regulate mitochondrion activity. The MAPK signaling pathway regulates apoptosis, and the context-dependent effects of MAPK signaling on apoptosis may be determined by specific MAPK subfamily members [27, 28]. To investigate the mechanism and validate the bioinformatic analysis findings, the expression of MAPK-related protein was analyzed via western blotting (Figure 4G). The OA group had significantly higher p-JNK/JNK, p-ERK/ERK, and p-P38/P38 ratios than the control group, as shown in Figure 4H–4J. However, ISO treatment significantly increased the levels of phosphorylation protein recovered. These findings showed that the ISO inhibited H_2_O_2_-induced oxidative stress by regulating MAPK signaling pathway.

### ISO protects chondrocytes from oxidative stress

### ISO decreases H2O2-induced ROS in chondrocytes

**Figure 5:**
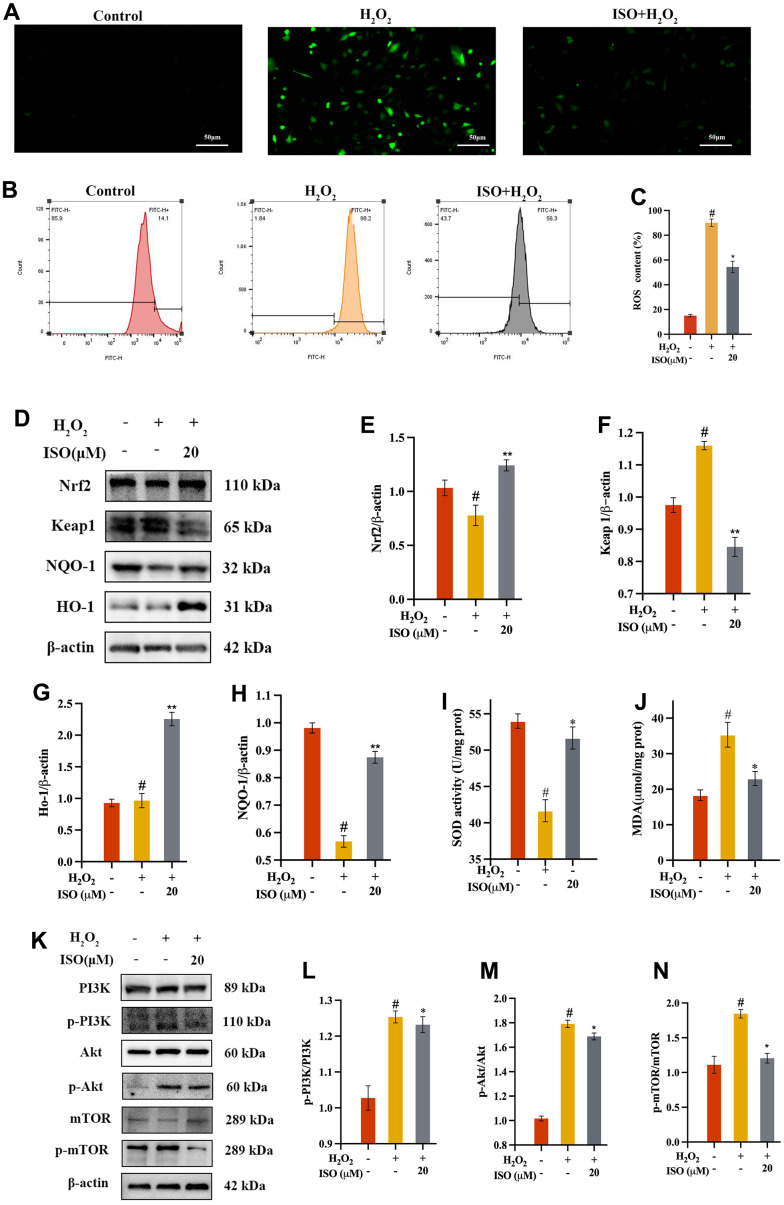
**Effect of ISO on oxidative stress.** (**A**) Fluorescence imaging analysis and (**B**) flow cytometry analysis on the ROS production of ISO treatment; (**C**) Bar graph summarizes the data on ROS production. (**D**–**H**) Effects of ISO pretreatment on the Nrf2/keap1 pathway by western blotting analysis. (**I**, **J**) Effect of ISO on the level of SOD and the activity of MDA of H_2_O_2_-induced injury Chondrocytes viability. (**K**–**N**) Effects of ISO pretreatment on the PI3K/Akt pathway by western blotting analysis. Results shown are expressed as means ± SD (n = 3). #p < 0.05 compared with control group, *p < 0.05 compared with OA group. The H_2_O_2_ concentration is 500 μM.

In order to further understand how ISO protect chondrocytes from apoptosis, we studied oxidative stress in this study. Apoptosis is typically caused by oxidative stress, which causes DNA damage and mitochondrial dysfunction [23, 25]. To validate the results of the bioinformatic analysis, we investigated ROS production in chondrocytes. DCFH-DA was used as a fluorescent probe to measure ROS production. The level of intracellular ROS may be indicated by DCF fluorescence [29]. As presented in Figure 5, the ROS were found using flow cytometry (Figure 5B) and an inverted fluorescence microscope (Figure 5A). H_2_O_2_ therapy significantly increased ROS production when compared to the control group. However, when compared to the OA group, ISO pretreatment significantly reduced ROS generation. The numerical outcome showed that ISO reduced H_2_O_2_-induced ROS overproduction (Figure 5C).

### ISO increases the expression of SOD, HO-1 and NQO-1 in chondrocytes

The increase in lipid peroxidation caused by ROS-induced oxidative damage typically reduces membrane fluidity and enzyme function [30]. The SOD level, which measures the strain on the intracellular oxidizing system, indirectly reflects the amount of intracellular free radicals [31]. When cells were treated with H_2_O_2_ alone, it was discovered that they had acute damage compared to the control group, as evidenced by a significant decrease in SOD. In contrast to the control group, incubating chondrocytes with ISO resulted in a significantly higher increase in SOD levels. (Figure 5I). After ISO pretreatment, antioxidant enzymes HO-1 and NQO-1 significantly increased (Figure 5G, 5H). The Nrf2/Keap1 signaling pathway is strongly linked to ISO oxidation resistance [32].

### ISO decreases H2O2-induced MDA production in chondrocytes

It is well known that MDA, a secondary byproduct of lipid peroxidation, is a marker of cell membrane damage. As a result, we determined the MDA content of the chondrocytes produced. The researchers found that MDA generation increased in response to 500 μM H_2_O_2_, thereby indicating cellular oxidative stress. However, MDA levels in the ISO group were lower than in the OA group (Figure 5D), indicating that ISO pretreatment reduced oxidative damage.

### ISO inhibits H2O2-induced oxidative stress by regulating Nrf2/keap1 and PI3K/Akt signaling pathways

Nrf2 regulates the endogenous antioxidant defense system. Nrf2 significantly increased after ISO pretreatment, as shown in Figure 5D, 5E. These findings suggested that the Nrf2 pathway may be involved in ISO’s control of H_2_O_2_-induced oxidative stress. Previous research showed that activated Akt phosphorylates affected the expression of Nrf2 downstream signaling pathway [7]. To confirm the bioinformatic analysis findings of ISO-treated H_2_O_2_-induced chondrocytes, we assessed the expression of the target protein of PI3K/Akt/mTOR signaling pathway through western blotting. According to ISO, the ratio of p-Akt to Akt has been restored, implying that the process by which ISO controls H_2_O_2_-induced oxidative stress may involve the PI3K/Akt/mTOR signaling pathway.

## DISCUSSION

Osteoarthritis, a chronic degenerative disease, is considered to affect patient health and quality of life [2]. According to recently published research, OA is an oxidative stress disease with an increased risk due to excessive oxidation [33, 34]. There are numerous treatments for OA, but none are conclusive. Herbal treatments are popular among researchers because they are non-toxic and have few side effects [35, 36]. ISO has a wide range of biological activities, such as antitumor and immunoregulatory activities. Furthermore, ISO has been reported in several publications to protect against oxidative damage [9, 21, 37]. However, few studies have been conducted on the protective mechanism of ISO. The articular cartilage and synovial fluid of osteoarthritis patients are subject to oxidative stress, so we simulated osteoarthritis *in vitro* by using H_2_O_2_ [38]. Our findings show that ISO protects cells from apoptosis and oxidative damage while having no toxic effect on rat chondrocytes (Figure 1C). It is a desire to seek and investigate highly efficient and low-side-effect biofunctional molecules to reduce oxidative damage, with the goal of further clarifying the mechanism of ISO for future application.

In recent years, RNA-seq and bioinformatics have emerged as new scientific research methods [39, 40]. RNA-seq and bioinformatics can provide valuable insights into a variety of aspects of an experiment [41]:1. RNA-seq and bioinformatics technologies can identify genes that are expressed differently in different groups or conditions, revealing details about their altered biological processes. 2. RNA-seq and bioinformatics technologies also provide data on alternative splicing events, allotypic use, and post-transcriptional modifications, allowing for a more comprehensive understanding of gene regulation. 3. RNA-seq and bioinformatics provide guidance for selecting genes for further validation using techniques like Western blotting and q-PCR. In the current study, ISO effectively prevents chondrocyte death. We used bioinformatics to sequence three groups of cells and analyze their differential genes. In our analysis, we discovered that ISO affects two biological processes, *apoptosis* and *oxidative stress*, so we validated them with laboratory tools.

Chondrocyte apoptosis (programmed cell death) is a critical factor in OA [42]. Apoptosis is thought to occur in the early stages of OA chondrocytes in order to protect them from mechanical stress and inflammation [43]. However, As the disease progresses, excessive chondrocyte apoptosis can destroy tissues and cartilage [44]. As a result, apoptosis inhibition may be useful in the treatment of OA. Reduced Bcl-2/Bax and increased cleaved caspase-3/caspase-3 levels are important indicators of mitochondrial apoptotic pathway activation. Our findings demonstrated that ISO could inhibit the expression of pro-apoptotic proteins induced by hydrogen peroxide as well as the reduction of apoptotic proteins in chondrocytes. Furthermore, the combination ISO treatment restored MMP in H_2_O_2_-induced chondrocytes. In brief, these findings suggest that ISO inhibits chondrocyte apoptosis induced by H_2_O_2_, which may be mediated by the MAPK signaling pathway. ISO has been shown in numerous studies to prevent apoptosis caused by the MAPK signaling pathway [27, 45–47]. This phenomenon is also confirmed in our current study, which provides a theoretical foundation for ISO osteoarthritis treatment.

Apoptosis and oxidative stress have been highly correlated in many studies [48, 49]. The imbalance between oxidation and antioxidation causes oxidative stress [11]. Currently, researchers are attempting to comprehend how ISO protects against oxidative stress. ROS is a key oxidative stress indicator that is involved in both physiological and pathological cell functions. According to Zheng et al., ISO protects human dermal fibroblasts (HDFs) from UVB-induced damage by lowering ROS generation [50]. This is consistent with our findings, and we also discovered that ISO can increase SOD activity and decrease MDA accumulation. Furthermore, Mina Alimohammadi et al. [51, 52] discovered that flavonoids (ISO is one of the flavonoids) increased SOD activity while decreasing MDA production. In conclusion, ISO may protect chondrocytes from oxidative injury by increasing SOD activity and decreasing ROS and MDA production. This antioxidant capacity has been demonstrated *in vitro* [53].

ISO protects against oxidative damage by increasing the activity of antioxidant enzymes and activating the Nrf2 antioxidant pathway [21, 54]. However, the protective mechanism of ISO against oxidative injury may be unclear in various disease models. The key signaling pathway that leads to antioxidant damage in organisms is known as Nrf2/Keap1. When exposed to oxidative stress, Nrf2 migrates to the nucleus and activates antioxidant response element (ARE) genes such as HO-1 and NQO-1. Western blot analysis revealed that ISO increased Nrf2 levels as well as HO-1 and NQO-1 protein expression, implying Nrf2/keap1 activation. In addition to validating the bioinformatics of KEGG, we also validated the PI3K/Akt pathway. Importantly, PI3K/Akt modulates Nrf2 activity primarily by phosphorylating Keap1, a negative regulator of Nrf2, and inhibiting its activity [5, 55]. Our findings showed that the PI3K/Akt pathway promotes Nrf2 activity and antioxidant gene expression, which can protect cells from oxidative stress and disease.

**Figure 6:**
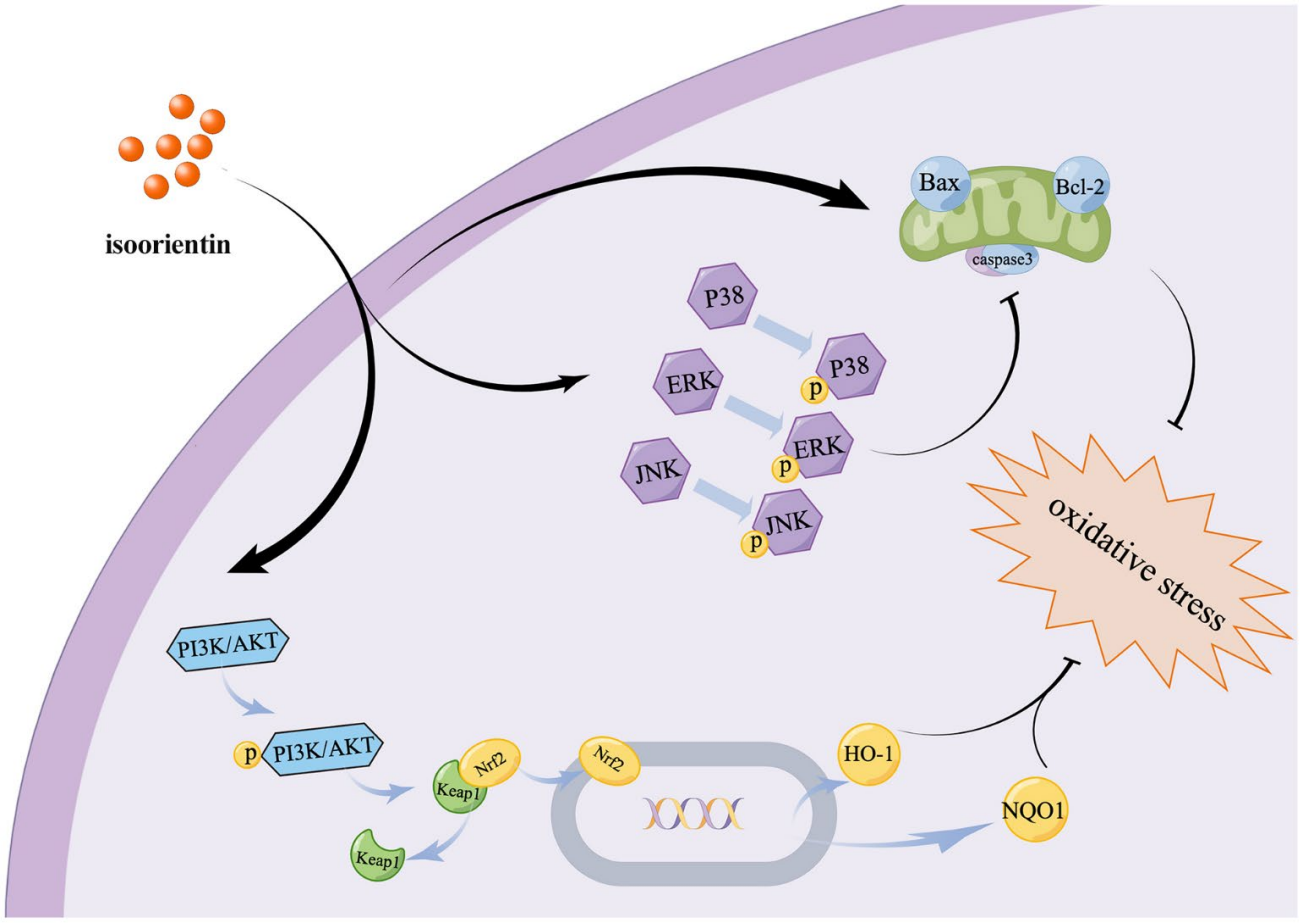
Diagram show the mechanism of ISO mitigates apoptosis and oxidative damage to Chondrocytes caused by H_2_O_2_.

Finally, the findings of this study indicate that ISO has the potential to relieve apoptosis and oxidative stress by modulating the MAPK and PI3K/Akt signaling pathways (Figure 6). This is the first study to examine the potential protective mechanisms of ISO in chondrocytes using RNA-seq and western blotting, providing insights into future directions for application through understanding ISO protective effects and signaling pathway targeting.

## CONCLUSIONS

In summary, OA has been proven to progress due to excessive oxidative stress. Increasing antioxidant levels may thus represent a novel strategy for slowing the progression of OA. Our findings highlight that ISO, as a novel antioxidant, can delay the progression of OA by inhibiting chondrocyte apoptosis and oxidative stress.
